# Supplementary material for: Structural insights into lipid chain-length selectivity and allosteric regulation of FFA2
Source: Nat Commun. 2025 Mar 26;16:2809. doi: 10.1038/s41467-025-57983-4 (PMC11947310; doi:10.1038/s41467-025-57983-4)
Supplement: Supplementary file 1 — Supplementary Information [file 41467_2025_57983_MOESM1_ESM.pdf]

# SUPPLEMENTARY INFORMATION

## Structural insights into lipid chain-length selectivity and allosteric regulation of FFA2


Mai Kugawa[1,2*], Kouki Kawakami[1*], Ryoji Kise[3*], Carl-Mikael Suomivuori[4,5,6,7], Masaki Tsujimura[8], Kazuhiro Kobayashi[1], Asato Kojima[1,9], Wakana J. Inoue[9], Masahiro Fukuda[1], Toshiki E. Matsui[1,9], Ayami Fukunaga[1,2], Junki Koyanagi[9], Suhyang Kim[1], Hisako Ikeda[1], Keitaro Yamashita[1], Keisuke Saito[1,10], Hiroshi Ishikita[1,10], Ron O. Dror[4,5,6,7], Asuka Inoue[3,11,12**] & Hideaki E. Kato[1,2,9,12,13**]

[1] Research Center for Advanced Science and Technology, The University of Tokyo, Meguro, Tokyo, Japan.

[2] Department of Biological Sciences, Graduate School of Science, The University of Tokyo, Bunkyo, Tokyo, Japan.

[3] Graduate School of Pharmaceutical Sciences, Tohoku University, Sendai, Japan.

[4] Department of Computer Science, Stanford University, Stanford, CA, USA.

[5] Department of Molecular and Cellular Physiology, Stanford University School of Medicine, Stanford, CA, USA.

[6] Department of Structural Biology, Stanford University School of Medicine, Stanford, CA, USA.

[7] Institute for Computational and Mathematical Engineering, Stanford University, Stanford, CA, USA.

[8] Department of Advanced Interdisciplinary Studies, The University of Tokyo, Meguro, Tokyo, Japan.

[9] Department of Life Sciences, School of Arts and Sciences, The University of Tokyo, Meguro, Tokyo, Japan.

[10] Department of Applied Chemistry, The University of Tokyo, Bunkyo, Tokyo, Japan.

[11] Graduate School of Pharmaceutical Sciences, Kyoto University, Kyoto, Japan.

[12] FOREST, Japan Science and Technology Agency, Kawaguchi, Saitama, Japan.

[13] CREST, Japan Science and Technology Agency, Kawaguchi, Saitama, Japan.

[*] These authors contributed equally: Mai Kugawa, Kouki Kawakami, Ryoji Kise.

[**]Corresponding authors: Asuka Inoue (iaska@tohoku.ac.jp) and Hideaki E. Kato (c-hekato@g.ecc.u-tokyo.ac.jp)

**Contents:**

**Supplementary Fig. 1:** Cryo-EM analysis of FFA2–Gi complex bound to TUG-1375 and 4-CMTB.

**Supplementary Fig. 2:** Cryo-EM analysis of FFA2-BRIL bound to GLPG0974.

**Supplementary Fig. 3:** Functional characterization of the orthosteric site.

**Supplementary Fig. 4:** Computational characterization of orthosteric ligand binding.

**Supplementary Fig. 5:** Computational characterization of antagonist binding.

**Supplementary Fig. 6:** Functional characterization of the allosteric ligand pocket.

**Supplementary Fig. 7:** Activation mechanism of FFA2.

**Supplementary Fig. 8:** Ligand entry and recognition in other lipid GPCRs.

**Supplementary Fig. 9:** Structural comparison of FFA2 and other GPCRs.

**Supplementary Fig. 10:** Comparison between experimentally determined and predicted FFA2 structures.

**Supplementary Fig. 11:** Water distribution in MD simulations.

**Supplementary Fig. 12:** MD simulation related to Supplementary Fig. 4.

**Supplementary Fig. 13:** Structural comparison of allosteric site 2 and three other reported FFA2 structures.

**Supplementary Table 1:** Cryo-EM data collection, refinement and validation statistics.

**Supplementary Table 2:** System setup for the MD simulations.

**Source Data: Uncropped gels for SDS-PAGE analysis.**

**References**

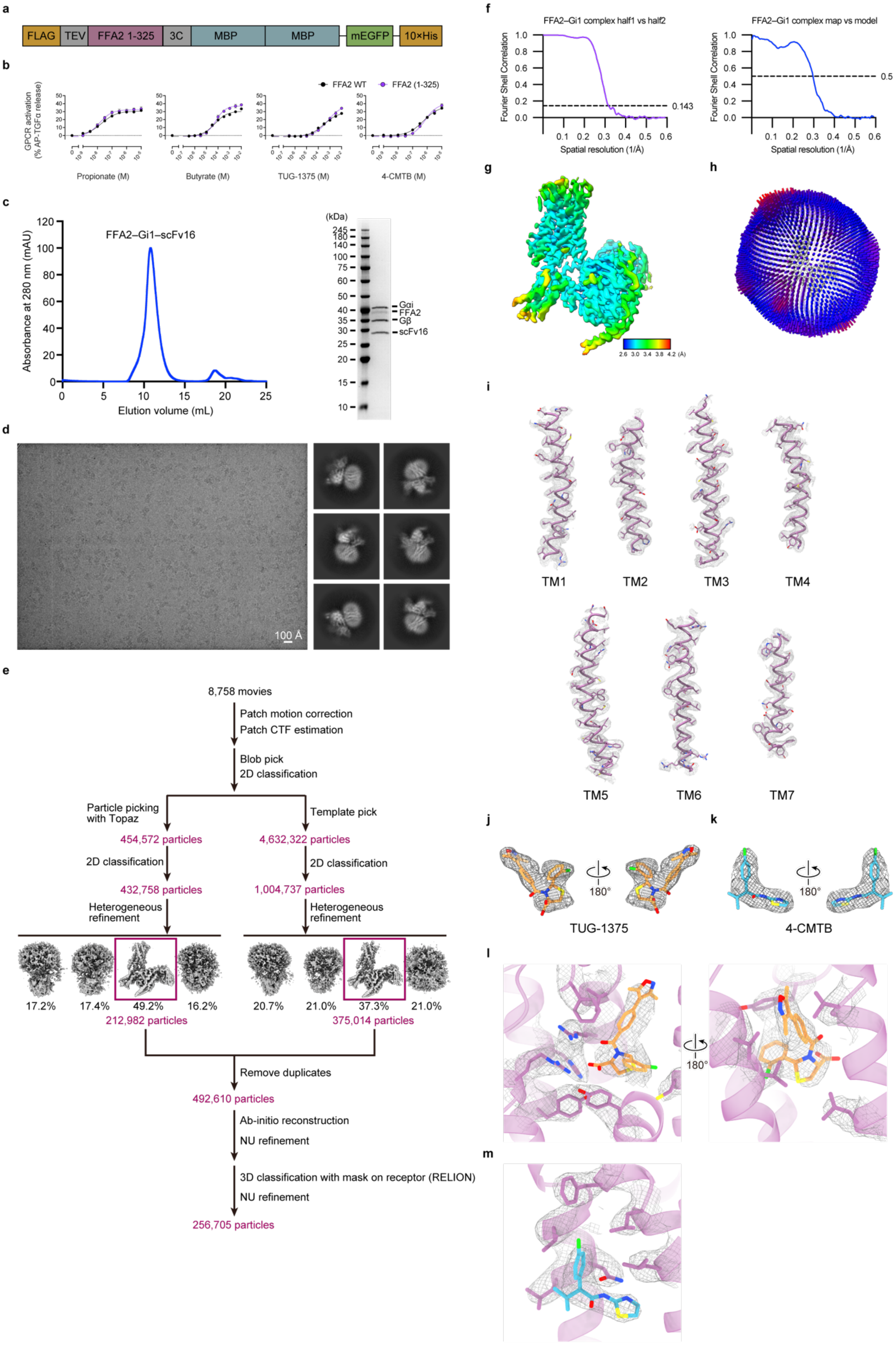
a
FLAG
TEV
FFA2 1-325
3C
MBP
MBP
mEGFP
10×His
b
FFA2 WT
FFA2 (1-325)
GPCR activation (% AP-TGFα release)
Propionate (M)
Butyrate (M)
TUG-1375 (M)
4-CMTB (M)
c
FFA2–Gi1–scFv16
Absorbance at 280 nm (mAU)
Elution volume (mL)
(kDa)
Gαi
FFA2
Gβ
scFv16
d
100 Å
e
8,758 movies
Patch motion correction
Patch CTF estimation
Blob pick
2D classification
Particle picking with Topaz
Template pick
454,572 particles
4,632,322 particles
2D classification
2D classification
432,758 particles
1,004,737 particles
Heterogeneous refinement
Heterogeneous refinement
17.2%
17.4%
49.2%
16.2%
20.7%
21.0%
37.3%
21.0%
212,982 particles
375,014 particles
Remove duplicates
492,610 particles
Ab-initio reconstruction
NU refinement
3D classification with mask on receptor (RELION)
NU refinement
256,705 particles
f
FFA2–Gi1 complex half1 vs half2
FFA2–Gi1 complex map vs model
Fourier Shell Correlation
Spatial resolution (1/Å)
0.143
0.5
g
2.6 3.0 3.4 3.8 4.2 (Å)
h
i
TM1
TM2
TM3
TM4
TM5
TM6
TM7
j
180°
TUG-1375
k
180°
4-CMTB
l
180°
m

**Supplementary Fig. 1: Cryo-EM analysis of FFA2–Gi complex bound to TUG-1375 and 4-CMTB.**

**a,** Schematic of the FFA2 construct used for protein expression and purification (see also Method section for details). **b,** Pharmacological activity of FFA2 (1-325) evaluated by the TGFα shedding assay upon stimulation with TUG-1375, propionate, butyrate and 4-CMTB. **c,** size-exclusion chromatography (SEC) trace (left) and SDS-PAGE analyses (right) of the FFA2–Gi complex bound to scFv16. **d,** A representative cryo-EM micrograph and 2D class averages. **e,** Data processing workflow. **f,** Fourier shell correlation (FSC) between the two independently refined half-maps (left) and between the model and the map calculated for the model refined against the full reconstruction (right). **g,** Local resolution analysis. **h,** Angular distribution of the particles used for the final reconstruction. **i-k,** Superimposed images of the cryo-EM density map and model for TM helices (**i**), TUG-1375 (**j**), and 4-CMTB (**k**). **l,m,** Superimposed images of the cryo-EM density maps and models showing TUG-1375 (**l**) and 4-CMTB (**m**) with their interacting residues in the binding pockets.

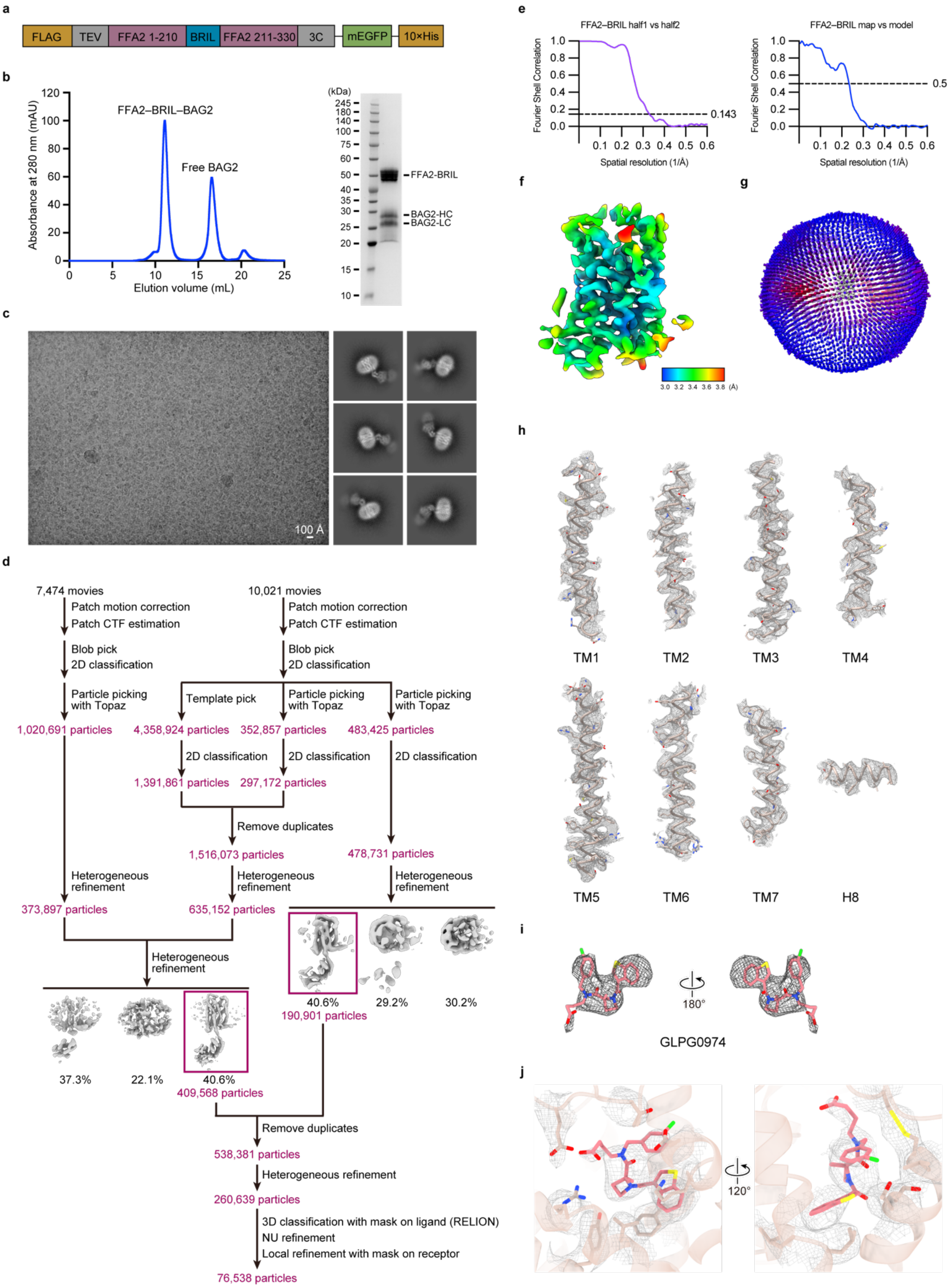
a
FLAG
TEV
FFA2 1-210
BRIL
FFA2 211-330
3C
mEGFP
10×His
b
FFA2–BRIL–BAG2
Free BAG2
Absorbance at 280 nm (mAU)
Elution volume (mL)
(kDa)
FFA2-BRIL
BAG2-HC
BAG2-LC
c
100 Å
d
7,474 movies
Patch motion correction
Patch CTF estimation
Blob pick
2D classification
Particle picking with Topaz
1,020,691 particles
10,021 movies
Patch motion correction
Patch CTF estimation
Blob pick
2D classification
Template pick
Particle picking with Topaz
Particle picking with Topaz
4,358,924 particles
352,857 particles
483,425 particles
2D classification
2D classification
2D classification
1,391,861 particles
297,172 particles
Remove duplicates
1,516,073 particles
478,731 particles
Heterogeneous refinement
Heterogeneous refinement
Heterogeneous refinement
373,897 particles
635,152 particles
Heterogeneous refinement
40.6%
190,901 particles
29.2%
30.2%
37.3%
22.1%
40.6%
409,568 particles
Remove duplicates
538,381 particles
Heterogeneous refinement
260,639 particles
3D classification with mask on ligand (RELION)
NU refinement
Local refinement with mask on receptor
76,538 particles
e
FFA2–BRIL half1 vs half2
FFA2–BRIL map vs model
Fourier Shell Correlation
Spatial resolution (1/Å)
0.143
0.5
f
g
3.0 3.2 3.4 3.6 3.8 (Å)
h
TM1
TM2
TM3
TM4
TM5
TM6
TM7
H8
i
180°
GLPG0974
j
120°

**Supplementary Fig. 2: Cryo-EM analysis of FFA2-BRIL bound to GLPG0974.**

**a,** Schematic of the BRIL-fused FFA2 construct (FFA2-BRIL) used in protein expression and purification (see also the Method section). **b,** SEC trace (left) and SDS-PAGE analyses (right) of FFA2-BRIL bound to anti-BRIL Fab (BAG2). **c,** A representative cryo-EM micrograph and 2D class averages. **d,** Data processing workflow. **e,** FSC between the two independently refined half-maps (left) and between the model and the map calculated for the model refined against the full reconstruction (right). **f,** Local resolution analysis. **g,** Angular distribution of the particles used for the final reconstruction. **h,i,** Cryo-EM density map for TM helices, helix 8, and ECL2 (**h**) and GLPG0974 (**i**). **j,** Superimposed images of the cryo-EM density map and model showing GLPG0974 (**j**) with their interacting residues in the binding pocket.

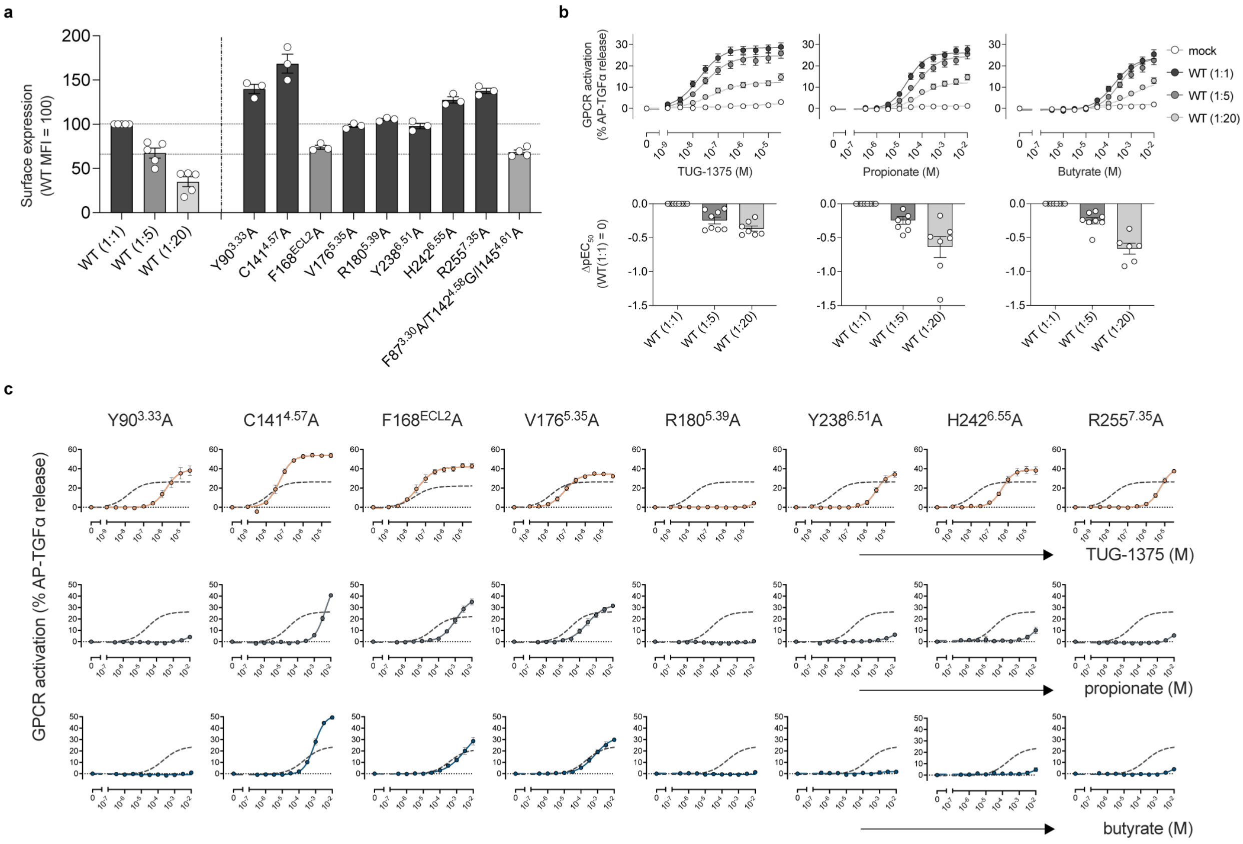


**Supplementary Fig. 3: Functional characterization of the orthosteric site.**

**a,** Cell surface expression level of the FFA2 mutants analyzed by flow cytometry. The mutants with similar expression levels to WT (1:1), (1:5), and (1:20) were colored black, dark gray, and light gray, respectively. **b,** Concentration-response curves (top) and $\Delta pEC_{50}$ values (bottom) of native FFA2 with distinct expression levels or mock-transfected cells upon stimulation with TUG-1375, propionate, and butyrate. **c,** Concentration-response curves of the FFA2 mutants upon stimulation with TUG-1375, propionate, and butyrate. Dashed lines in each panel indicate the responses of WT with similar expression levels. In panels **a** and **b,** bars and error bars represent mean and SEM, respectively. In **b** and **c,** symbols and error bars represent mean and SEM, respectively. All of these data were derived from 3-8 independent experiments, each performed in duplicate. ** represent $p < 0.01$, with one-way ANOVA followed by Dunnett's test for multiple comparison analysis with reference to WT. ns, not significantly different between the groups.

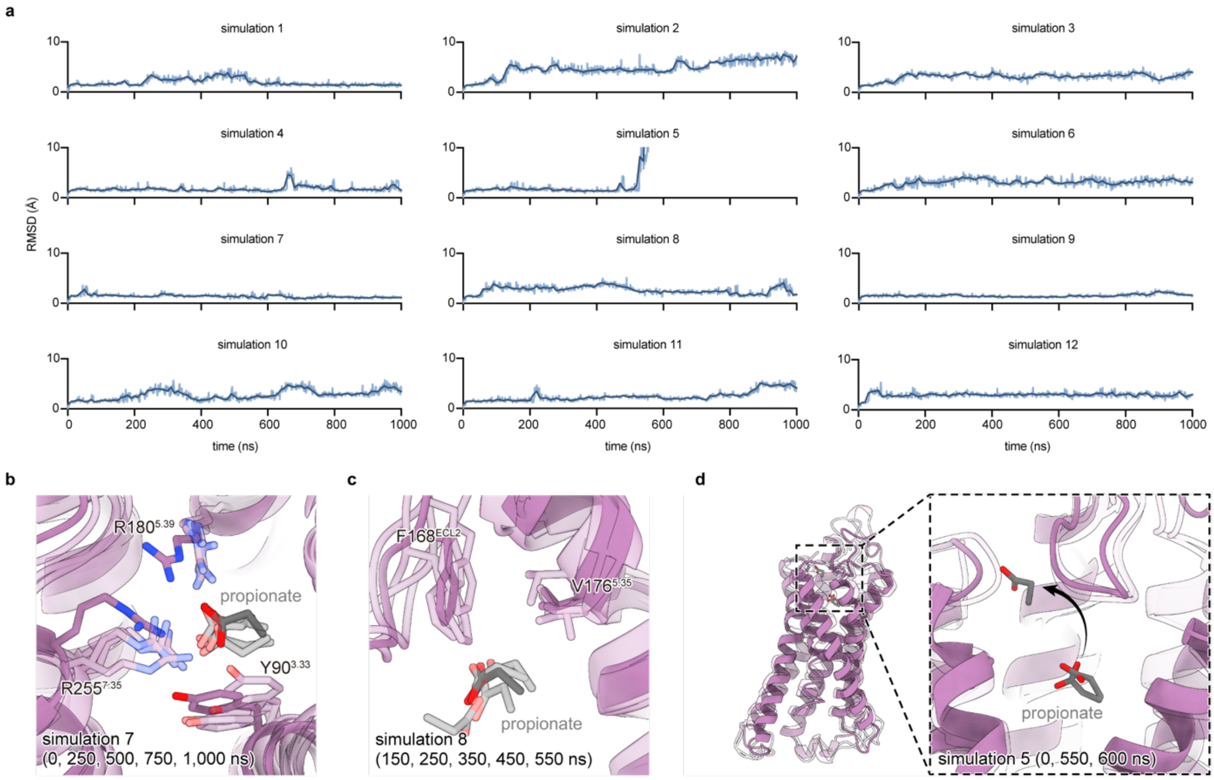


**Supplementary Fig. 4: Computational characterization of orthosteric ligand binding.**

**a,** Root-mean-square deviation (RMSD) of propionate from its initial position, after aligning on protein backbone atoms, during 12 independent simulations. Shaded lines represent unsmoothed values, while solid lines represent a moving average using a smoothing window of 10 ns. Note that in simulation 5, propionate dissociates and enters the extracellular solvent. **b,** Representative frames (at 0, 250, 500, 750, and 1,000 ns) from simulation 7, in which propionate remains stable in its initial pose, interacting with Y90$^{3.33}$, R180$^{5.39}$, and R255$^{7.35}$. **c,** Representative frames (at 0, 250, 500, 750, and 1,000 ns) from simulation 8, in which propionate loses its initial interactions and forms transient new interactions with surrounding residues, including F168$^{ECL2}$ and V176$^{5.35}$. **d,** Frames at 0, 550, and 600 ns in simulation 5, in which propionate dissociates and enters the extracellular solvent. The black arrow indicates movement of propionate during the simulation.

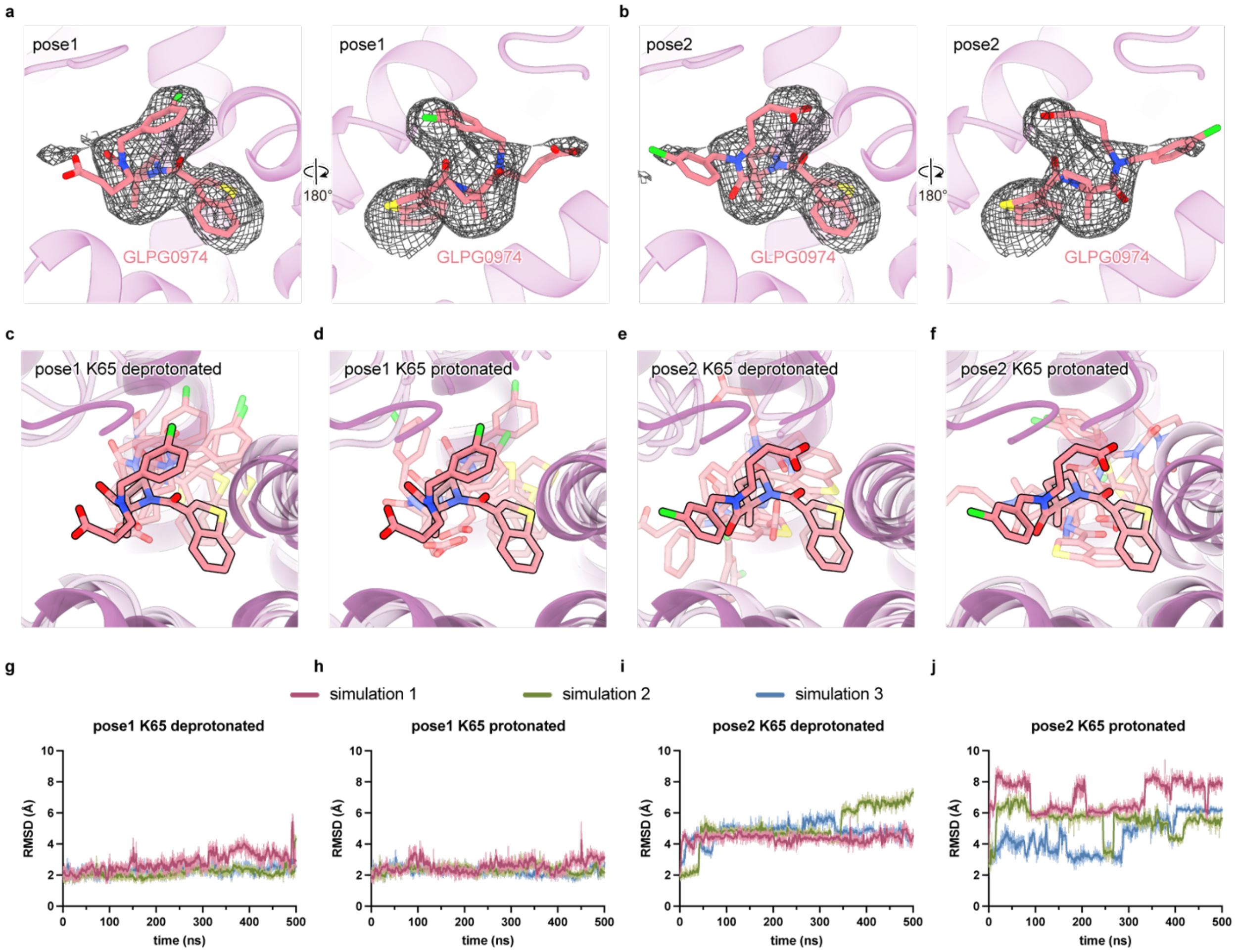


k

| | 2.56 | 2.60 | 2.63 | ECL1 | 3.25 | 3.28 | 3.29 | 3.32 | ECL2 | ECL2 | ECL2 | 6.51 | 7.35 | 7.39 |
|---|---|---|---|---|---|---|---|---|---|---|---|---|---|---|
| FFA2 | L | K | E | L | C | T | S | F | T | C | E | Y | R | V |
| FFA3 | F | R | - | L | C | S | G | F | N | C | L | Y | R | T |

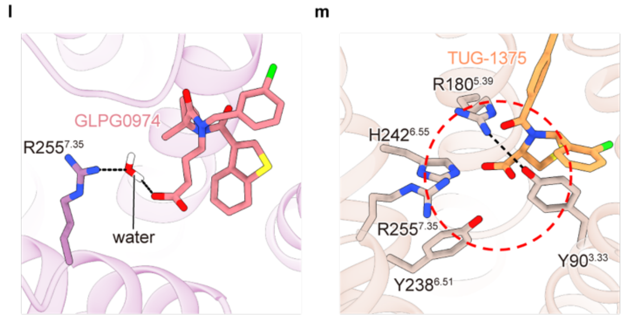


**Supplementary Fig. 5: Computational characterization of antagonist binding.**

**a,b,** Cryo-EM density map and model focused on GLPG0974 in pose 1 (**a**) and pose 2 (**b**). **c-f,** MD simulations of GLPG0974 in inactive FFA2. The solid sticks show the GLPG0974 structure in the cryo-EM model. The shaded sticks show the GLPG0974 structure in the final snapshots taken at 500 ns from each of the three simulation replicates.

**g-j,** RMSD values of GLPG0974 atoms with respect to the cryo-EM model in the three independent MD simulations (Å). The shaded lines represent unsmoothed values, while the solid lines represent a moving average using a smoothing window of 1 ns. **k,** Sequence comparison of GLPG0974-binding sites at FFA2 and FFA3. **l,** Snapshot at 300 ns from simulation 1 with pose1 GLPG0974 and protonated Lys65$^{2.60}$. **m,** A docked model of inactive state FFA2 with TUG-1375. The red dashed circle indicates the steric clash between inactive-state FFA2 and TUG-1375. In **l** and **m,** hydrogen bonds are represented as dashed black lines.

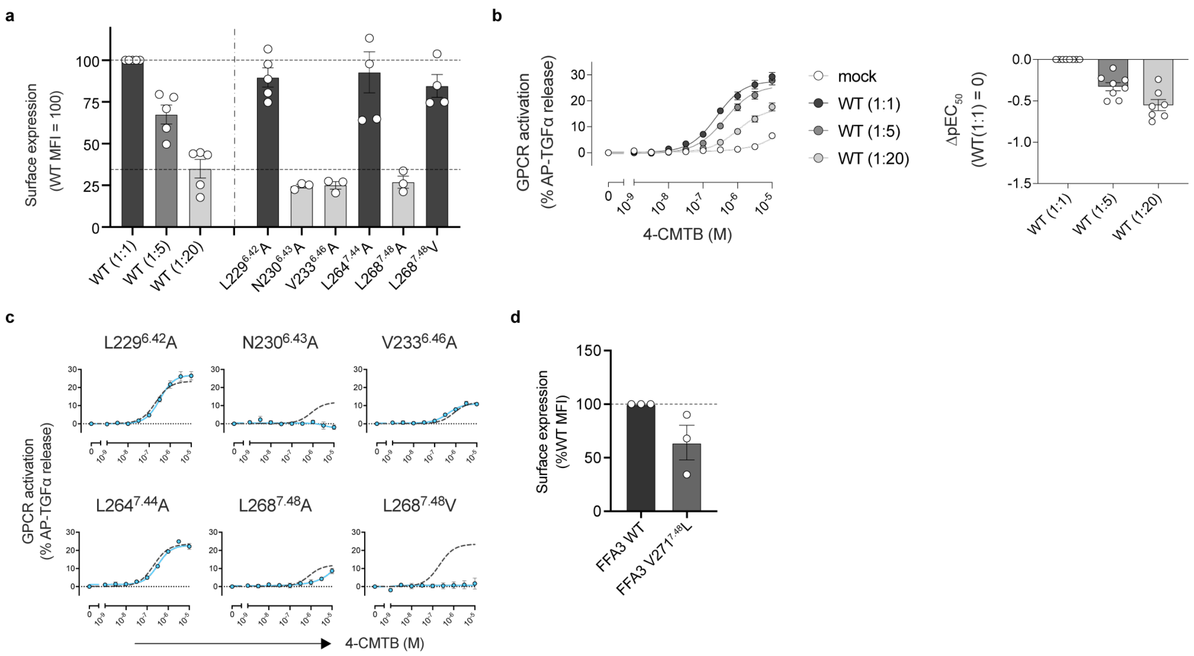


**Supplementary Fig. 6: Functional characterization of the allosteric ligand pocket.**

**a,** Cell surface expression level of the FFA2 mutants analyzed by flow cytometry. The mutants with similar expression levels to WT (1:1), (1:5), and (1:20) were colored black, dark gray, and light gray, respectively. Bars and error bars represent mean and SEM, respectively, of 3-5 independent experiments, each performed in duplicate. **b,** Concentration-response curves (top) and $\Delta pEC_{50}$ values (bottom) of native FFA2 with distinct expression levels or mock-transfected cells upon stimulation with 4-CMTB. **c,** Concentration-response curves of the FFA2 mutants. Dashed lines in each panel indicate the responses of FFA2-WT with similar expression levels. In **b** and **c,** symbols and error bars represent mean and SEM, respectively. All of these data were derived from 3-8 independent experiments, each performed in duplicate. **d,** Cell surface expression level of the FFA3 mutant analyzed by flow cytometry. Bars and error bars represent mean and SEM, respectively, of three independent experiments, each performed in duplicate.

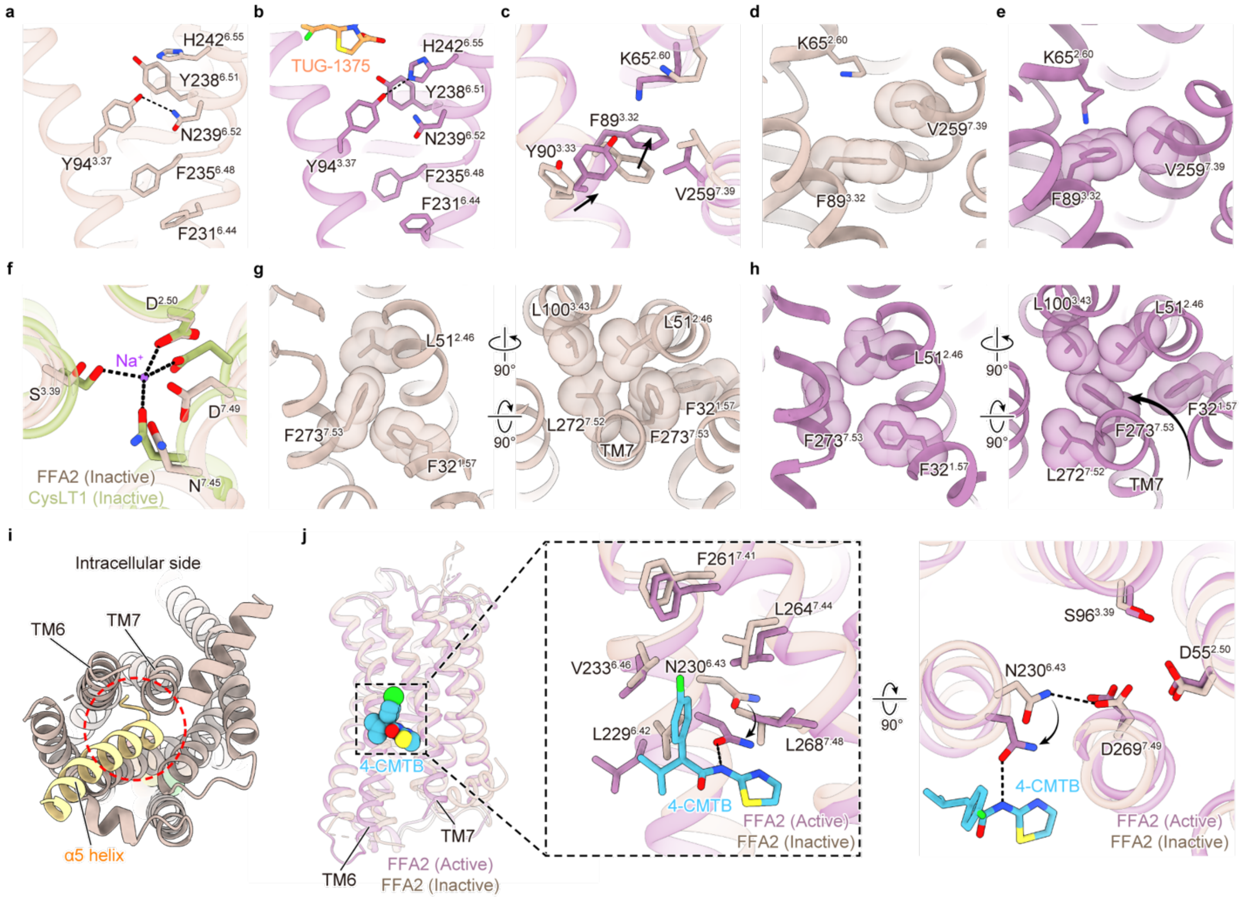


**Supplementary Fig. 7: Activation mechanism of FFA2.**

**a,b,** The extracellular side of TM3-6 interface in inactive (**a**) and active (**b**) FFA2. The regions of the views correspond to Fig. 6c. Hydrogen bonds are represented as dashed black lines. **c,** Superimposed image of active FFA2 (purple) and inactive FFA2 (beige) focused on the conformational changes of F89$^{3.32}$ and Y90$^{3.33}$. **d,e,** The interaction network between K65$^{2.60}$, F89$^{3.32}$, and V259$^{7.39}$ in inactive (**d**) and active (**e**) FFA2. **f,** Superimposed image of the sodium binding site of CysLT1[91] (PDB ID: 6RZ5) and that of inactive FFA2. The purple sphere represents the sodium ion observed in the CysLT1 structure. Ionic interactions are represented as dashed black lines. **g,h,** The packing of TM7 against TMs 1-3 in inactive (**g**) and active (**h**) FFA2. The black arrow indicates the conformational change of F273$^{7.53}$. **i,** A docked model of inactive FFA2 (beige) and the α5 helix (yellow) of the FFA2–Gi complex. The red dashed circle indicates the steric clash between inactive FFA2 and the α5 helix. **j,** Superimposed image of the overall structure of FFA2 bound to 4-CMTB and inactive FFA2 (left), an enlarged view of the allosteric ligand pocket

focused on 4-CMTB (center, right). Hydrogen bonds and the conformational change of N230$^{6.43}$ are represented as dashed black lines and a solid black arrow, respectively.

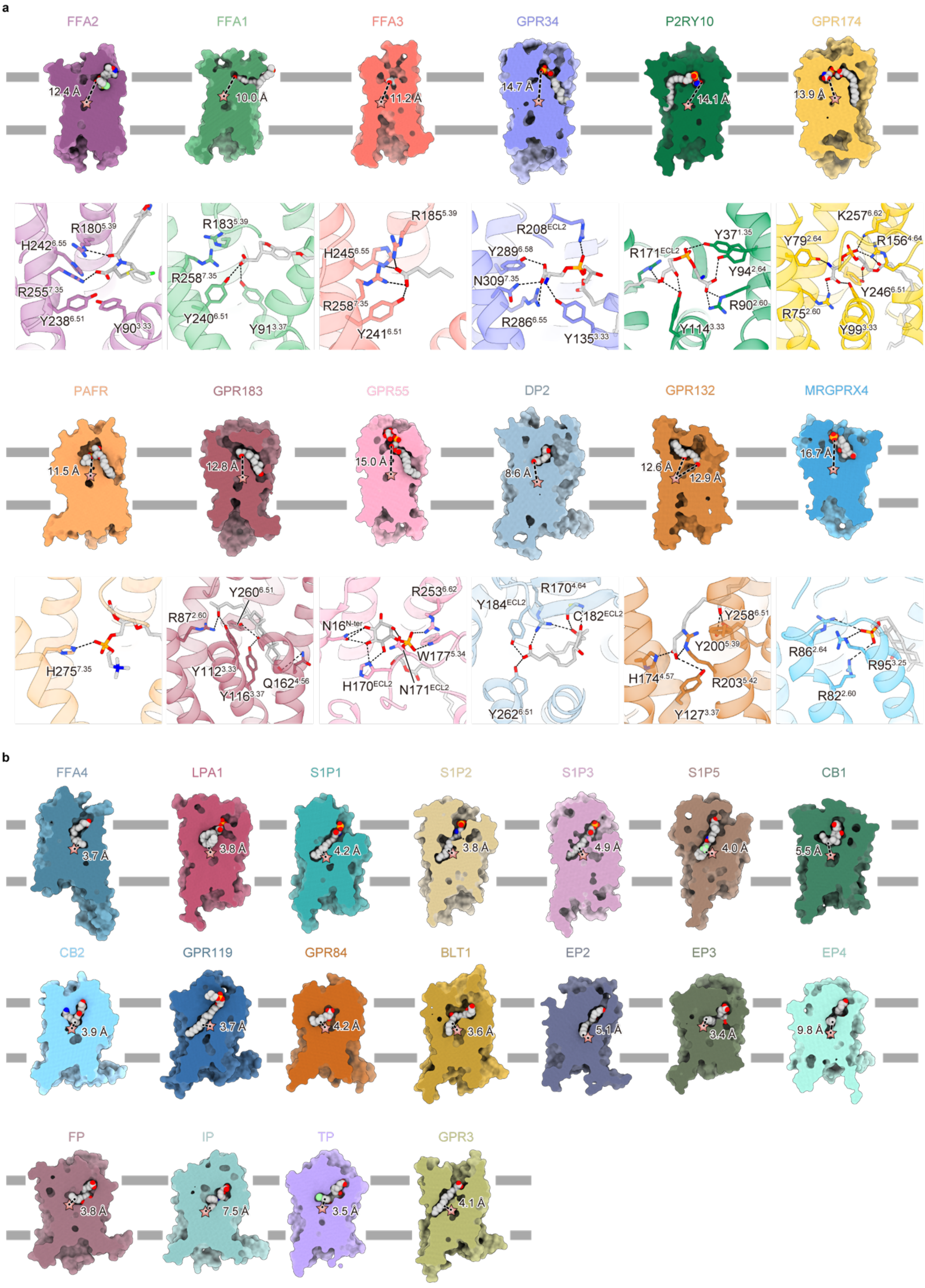

a
FFA2
FFA1
FFA3
GPR34
P2RY10
GPR174
12.4 Å
10.0 Å
11.2 Å
14.7 Å
14.1 Å
13.9 Å
R180$^{5.39}$
H242$^{6.55}$
R255$^{7.35}$
Y238$^{6.51}$
Y90$^{3.33}$
R183$^{5.39}$
R258$^{7.35}$
Y240$^{6.51}$
Y91$^{3.37}$
R185$^{5.39}$
H245$^{6.55}$
R258$^{7.35}$
Y241$^{6.51}$
R208$^{ECL2}$
Y289$^{6.58}$
N309$^{7.35}$
R286$^{6.55}$
Y135$^{3.33}$
Y371$^{1.35}$
R171$^{ECL2}$
Y94$^{2.64}$
R90$^{2.60}$
Y114$^{3.33}$
K257$^{6.62}$
Y79$^{2.64}$
R156$^{4.64}$
Y246$^{6.51}$
R75$^{2.60}$
Y99$^{3.33}$
PAFR
GPR183
GPR55
DP2
GPR132
MRGPRX4
11.5 Å
12.8 Å
15.0 Å
8.6 Å
12.6 Å
12.9 Å
16.7 Å
H275$^{7.35}$
Y260$^{6.51}$
R87$^{2.60}$
Y112$^{3.33}$
Q162$^{4.56}$
Y116$^{3.37}$
R253$^{6.62}$
N16$^{N-ter}$
W177$^{5.34}$
H170$^{ECL2}$
N171$^{ECL2}$
R170$^{4.64}$
Y184$^{ECL2}$
C182$^{ECL2}$
Y262$^{6.51}$
Y258$^{6.51}$
Y200$^{5.39}$
H174$^{4.57}$
R203$^{5.42}$
Y127$^{3.37}$
R86$^{2.64}$
R95$^{3.25}$
R82$^{2.60}$
b
FFA4
LPA1
S1P1
S1P2
S1P3
S1P5
CB1
3.7 Å
3.8 Å
4.2 Å
3.8 Å
4.9 Å
4.0 Å
5.5 Å
CB2
GPR119
GPR84
BLT1
EP2
EP3
EP4
3.9 Å
3.7 Å
4.2 Å
3.6 Å
5.1 Å
3.4 Å
9.8 Å
FP
IP
TP
GPR3
3.8 Å
7.5 Å
3.5 Å
4.1 Å

**Supplementary Fig. 8: Ligand entry and recognition in other lipid GPCRs.**

**a,** Cross-section representation (top) and ligand-binding site focused on the ligand's polar group (bottom) of TUG-1375-bound FFA2 (this study), TAK-875-bound FFA1[24] (PDB ID: 8EJC), valeric acid-bound FFA3[53] (PDB ID: 8J20), LysoPS-bound GPR34[92] (PDB ID: 8SAI), LysoPS-bound P2Y10[93] (PDB ID: 8KGG), LysoPS-bound GPR174[94] (PDB ID: 7XV3), PAF-bound PAFR[95] (PDB ID: 8XYD), 7α,25-dihydroxycholesterol-bound GPR183[96] (PDB ID: 7TUZ), LPI-bound GPR55[97] (PDB ID: 8ZX4), 15R-methyl-PGD2-bound DP2[98] (PDB ID: 7M8W), 9(S)-HODE-bound GPR132[99] (PDB ID: 8HQN), and DCA-3P-bound MRGPRX4[100] (PDB ID: 8K4S). **b,** Cross-section representation of EPA-bound FFA4[38] (PDB ID: 8ID9), ONO-9780307-bound LPA1[59] (PDB ID: 7TD0), S1P-bound S1P1[101] (PDB ID: 7VIE), S1P-bound S1P2[102] (PDB ID: 7T6B), S1P-bound S1P3[103] (PDB ID: 7EW3), Siponimod-bound S1P5[104] (PDB ID: 7EW1), CP55940-bound CB1[51] (PDB ID: 7WV9), AM12033-bound CB2[105] (PDB ID: 6KPF), LPC-bound GPR119[106] (PDB ID: 7XZ5), 3-OH-C12-bound GPR84[107] (PDB ID: 8J18), LTB4-bound BLT1[108] (PDB ID: 7VKT), PGE2-bound EP2[109] (PDB ID: 7CX2), PGE2-bound EP3[110] (PDB ID: 8GDC), PGE2-bound EP4[111] (PDB ID: 7D7M), PGF2α-bound FP[112] (PDB ID: 8IUK), MRE-269-bound IP[113] (PDB ID: 8X79), Cloprosetnol-bound TP[114] (PDB ID: 8XJN), and oleic acid-bound GPR3[115] (PDB ID: 8WW2). In all panels, carbon, oxygen, nitrogen, and phosphorus atoms of each ligand are colored in gray, red, blue, and yellow, respectively. The stars in the middle of each receptor represent the position of the residues at 6.48 in the BW numbering. The dashed lines represent the distance between the residues at 6.48 and the closest atoms of a polar group (**a**) or hydrocarbon chain (**b**) of each ligand.

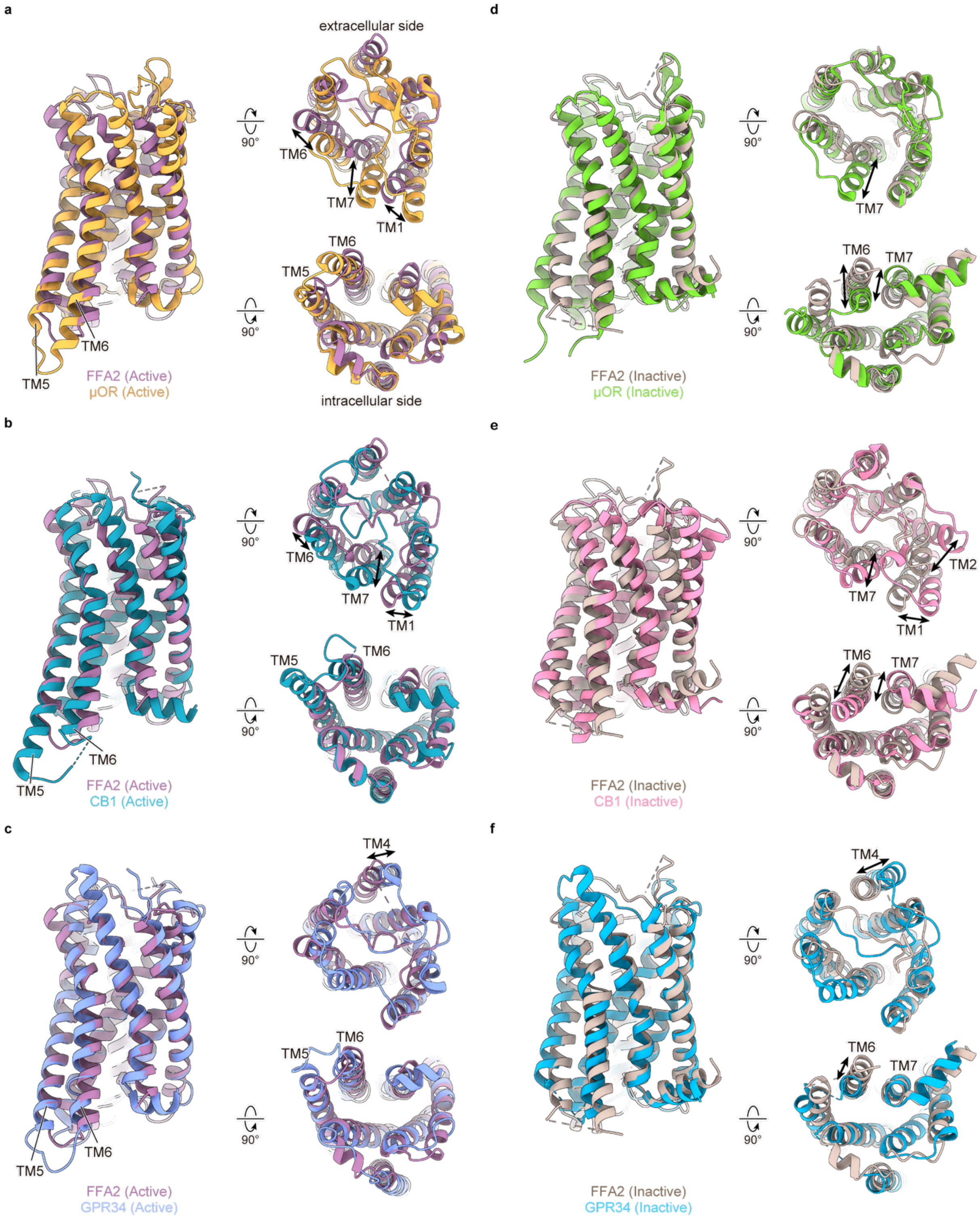


**Supplementary Fig. 9: Structural comparison of FFA2 and other GPCRs.**

**a-c,** Superimposed images of the active FFA2 structure with μOR (**a**, 5C1M), CB1 (**b**, 7WV9), and GPR34 (**c**, 8SAI).

**d-f,** Superimposed images of the inactive FFA2 structure with μOR (**d**, 4DKL), CB1 (**e**, 5U09), and GPR34 (**f**, 8IYX).

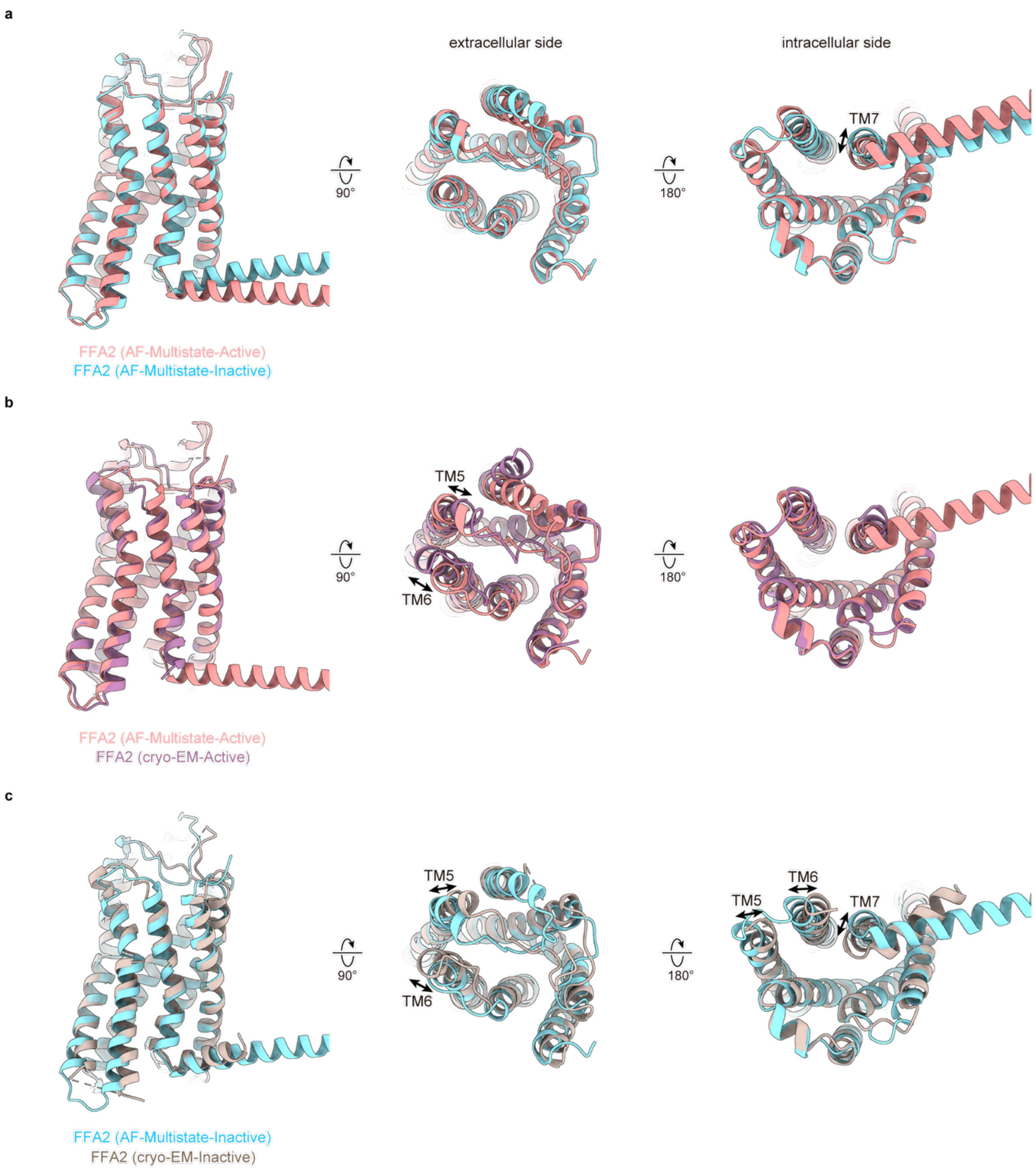


**Supplementary Fig. 10: Comparison between experimentally determined and predicted FFA2 structures.**

**a,** Superimposed image of predicted active and inactive FFA2 structures obtained from GPCRdb. Both inactive (cyan) and active (pink) structures were predicted by AlphaFold-Multistate (AF-Multistate). **b,** Superimposed image of the cryo-EM (purple) and predicted (pink) FFA2 structures in the active state. **c,** Superimposed image of the cryo-EM (beige) and predicted (cyan) FFA2 structures in the inactive state.

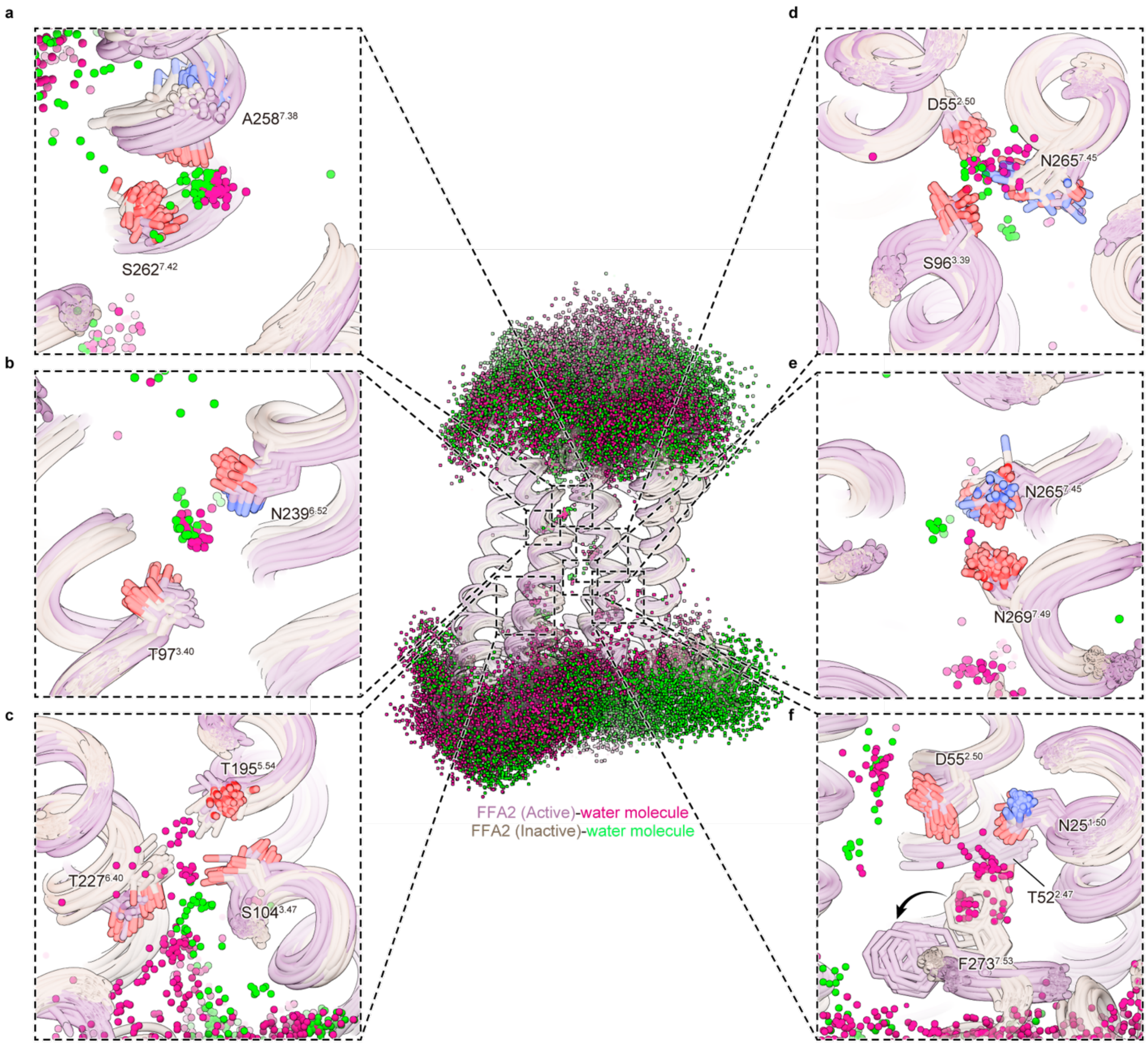


**Supplementary Fig. 11: Water distribution in MD simulations.**

**a–f,** Water distribution sampled every 50 ns from 50 to 500 ns in the three simulations for the active and inactive (with deprotonated K65) state receptors. Water clusters observed between A258$^{7.38}$ and S262$^{7.42}$ (**a**), T97$^{3.40}$ and N239$^{6.52}$ (**b**), S104$^{3.47}$, T195$^{5.54}$, and T227$^{6.40}$ (**c**), D55$^{2.50}$, S96$^{3.39}$, and N265$^{7.45}$ (**d**), N265$^{7.45}$ and N269$^{7.49}$ (**e**), and N25$^{1.50}$, T52$^{2.47}$, D55$^{2.50}$, and F273$^{7.53}$ (**f**) are enlarged in each dashed rectangle. Water molecules observed in active-state simulations are represented as magenta spheres, while those observed in inactive-state simulations are represented as green spheres. Receptor structures in the active and inactive states are depicted as semi-transparent cartoons and sticks, and are colored in purple and beige, respectively. The black arrow in (**f**) represents the movement of F273$^{7.53}$ upon receptor activation.

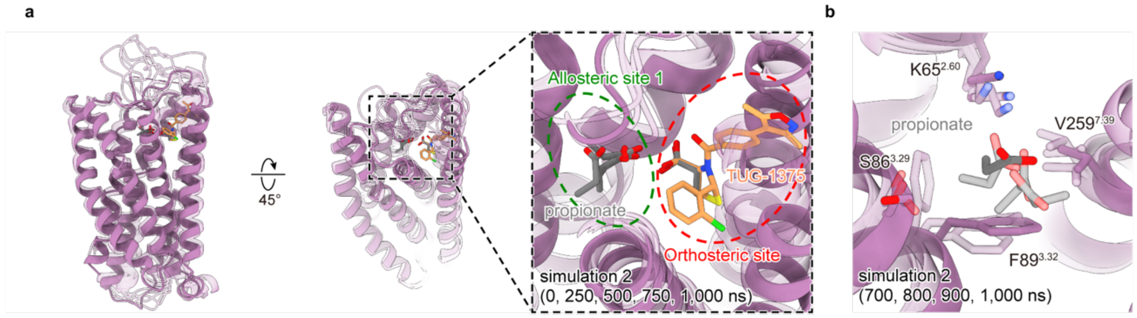


**Supplementary Fig. 12: MD simulation related to Supplementary Fig. 4.**

**a,b,** Representative simulation frames from simulation 2 of propionate-bound FFA2 at 0, 250, 500, 750, and 1,000 ns (a), and at 700, 800, 900, and 1,000 ns (b). The red and green dashed circles indicate the orthosteric site and allosteric site 1, respectively.

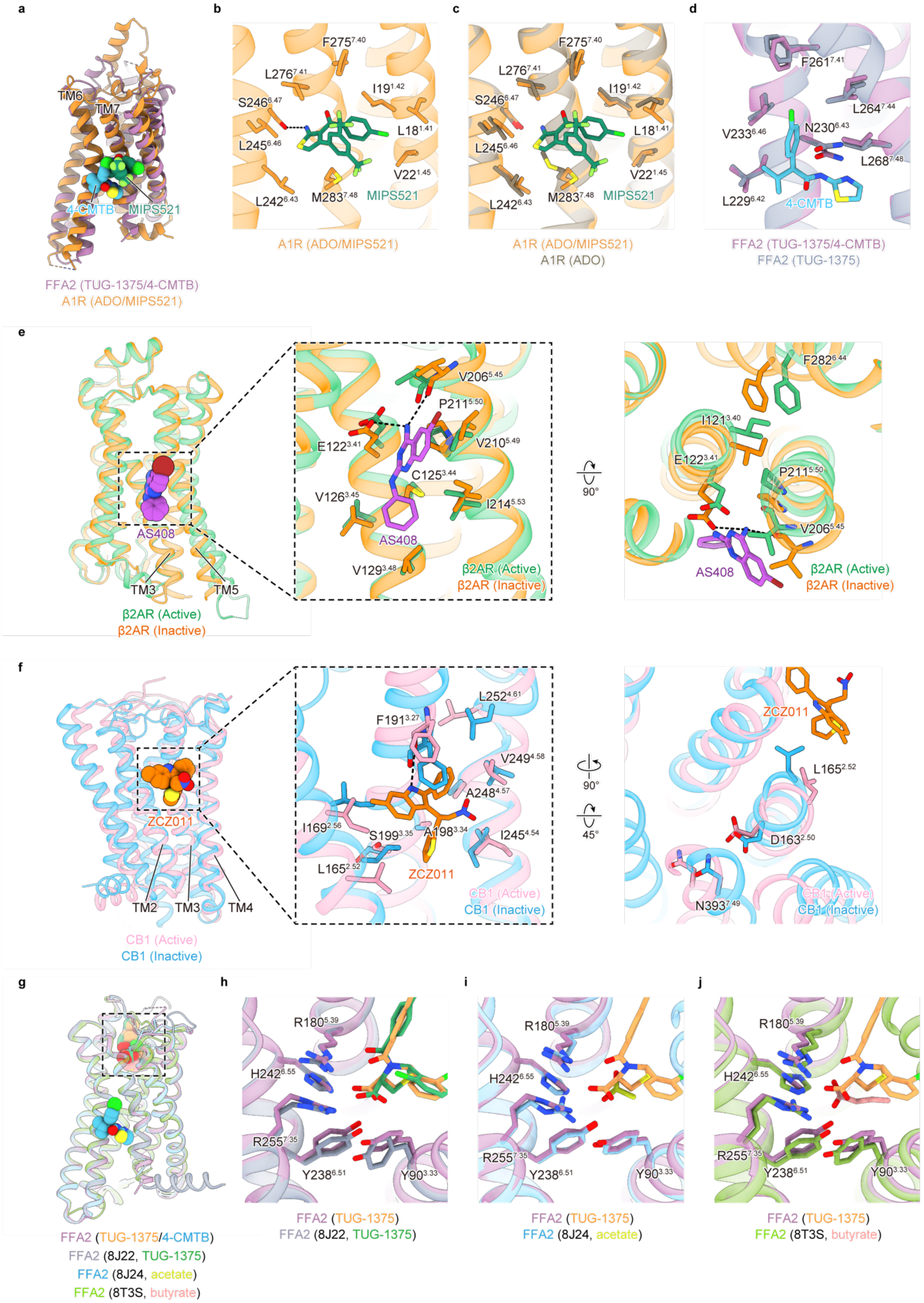
a
TM6
TM7
4-CMTB
MIPS521
FFA2 (TUG-1375/4-CMTB)
A1R (ADO/MIPS521)
b
F275$^{7.40}$
L276$^{7.41}$
I19$^{1.42}$
S246$^{6.47}$
L18$^{1.41}$
L245$^{6.46}$
V22$^{1.45}$
L242$^{6.43}$
M283$^{7.48}$
MIPS521
A1R (ADO/MIPS521)
c
F275$^{7.40}$
L276$^{7.41}$
I19$^{1.42}$
S246$^{6.47}$
L18$^{1.41}$
L245$^{6.46}$
V22$^{1.45}$
L242$^{6.43}$
M283$^{7.48}$
MIPS521
A1R (ADO/MIPS521)
A1R (ADO)
d
F261$^{7.41}$
L264$^{7.44}$
V233$^{6.46}$
N230$^{6.43}$
L268$^{7.48}$
L229$^{6.42}$
4-CMTB
FFA2 (TUG-1375/4-CMTB)
FFA2 (TUG-1375)
e
AS408
TM3
TM5
β2AR (Active)
β2AR (Inactive)
V206$^{5.45}$
P211$^{5.50}$
E122$^{3.41}$
V210$^{5.49}$
C125$^{3.44}$
V126$^{3.45}$
I214$^{5.53}$
AS408
V129$^{3.48}$
β2AR (Active)
β2AR (Inactive)
90°
F282$^{6.44}$
I121$^{3.40}$
E122$^{3.41}$
P211$^{5.50}$
V206$^{5.45}$
AS408
β2AR (Active)
β2AR (Inactive)
f
ZCZ011
TM2
TM3
TM4
CB1 (Active)
CB1 (Inactive)
L252$^{4.61}$
F191$^{3.27}$
V249$^{4.58}$
A248$^{4.57}$
I169$^{2.56}$
S199$^{3.35}$
A198$^{3.34}$
I245$^{4.54}$
L165$^{2.52}$
ZCZ011
CB1 (Active)
CB1 (Inactive)
90°
45°
ZCZ011
L165$^{2.52}$
D163$^{2.50}$
N393$^{7.49}$
CB1 (Active)
CB1 (Inactive)
g
FFA2 (TUG-1375/4-CMTB)
FFA2 (8J22, TUG-1375)
FFA2 (8J24, acetate)
FFA2 (8T3S, butyrate)
h
R180$^{5.39}$
H242$^{6.55}$
R255$^{7.35}$
Y238$^{6.51}$
Y90$^{3.33}$
FFA2 (TUG-1375)
FFA2 (8J22, TUG-1375)
i
R180$^{5.39}$
H242$^{6.55}$
R255$^{7.35}$
Y238$^{6.51}$
Y90$^{3.33}$
FFA2 (TUG-1375)
FFA2 (8J24, acetate)
j
R180$^{5.39}$
H242$^{6.55}$
R255$^{7.35}$
Y238$^{6.51}$
Y90$^{3.33}$
FFA2 (TUG-1375)
FFA2 (8T3S, butyrate)

**Supplementary Fig. 13: Structural comparison of allosteric site 2 and three other reported FFA2 structures.** **a,** Superimposed image of FFA2 bound to 4-CMTB (purple, cyan) and A1R bound to MIPS521[48] (orange, green) (PDB ID: 7LD3). **b,** MIPS521-bound allosteric pocket of A1R. Hydrogen bond is represented as a dashed black line. **c,** Comparison of the allosteric pocket of A1R in complex with both adenosine (ADO) and MIPS521[48] (orange) (PDB ID: 7LD3) and with only ADO (brown) (PDB ID: 7LD4). **d,** Comparison of the allosteric site 2 of FFA2 in complex with both TUG-1375 and 4-CMTB (purple) and with only TUG-1375[53] (gray) (PDB ID: 8J22). **e,** Superimposed image of active β2AR (green) (PDB ID: 4LDO) and inactive β2AR bound to AS408 (orange, purple) (PDB ID: 6OBA). **f,** Superimposed image of active CB1 bound to ZCZ011 (pink, orange) (PDB ID: 7WV9) and inactive CB1 (light blue) (PDB ID: 5U09). **g**, Superimposed image of FFA2 structure in this study and three other reported FFA2 structures (PDB ID: 8J22, 8J24, 8T3S). **h**, Superimposed image of TUG-1375/4-CMTB-bound FFA2 (this study) and TUG-1375-bound FFA2 (PDB ID: 8J22), focused on the TUG-1375-binding site. **i,** Superimposed image of TUG-1375/4-CMTB-bound FFA2 (this study) and acetate-bound FFA2 (PDB ID: 8J24), focused on the TUG-1375-binding site. **j,** TUG-1375/4-CMTB-bound FFA2 (this study) and butyrate-bound FFA2 (PDB ID: 8T3S), focused on the TUG-1375-binding site.

**Supplementary Table 1:** Cryo-EM data collection, refinement and validation statistics.

| | FFA2-Gi complex (EMDB-39003) (PDB 8Y6W) | FFA2-BRIL (EMDB-39004) (PDB 8Y6Y) | |
|---|---|---|---|
| **Data collection and processing** | | | |
| Magnification | 105,000 | 105,000 | 105,000 |
| Voltage (kV) | 300 | 300 | 300 |
| Electron exposure (e–/Å$^2$) | 45.6 | 45.6 | 57.9 |
| Defocus range (µm) | -0.8 to -1.6 | -0.8 to -1.6 | -0.8 to -1.6 |
| Pixel size (Å) | 0.83 | 0.83 | 0.83 |
| Initial collected movies (no.) | 8,758 | 7,474 | 10,021 |
| Symmetry imposed | C1 | C1 | |
| Initial particle images (no.) | 4,703,440 | 5,581,238 | |
| Final particle images (no.) | 256,705 | 76,538 | |
| Map resolution (Å) | 3.19 | 3.36 | |
| FSC threshold | 0.143 | 0.143 | |
| | | | |
| **Refinement** | | | |
| Initial model used (PDB code) | AF2 model for FFA2<br>7TD4 for Gi | AF2 model | |
| Model composition | | | |
| Non-hydrogen atoms | 8,750 | 2,186 | |
| Protein residues | 1109 | 278 | |
| Ligands | 2 | 1 | |
| *B* factors (Å$^2$) | | | |
| Protein | 78.4 | 40.5 | |
| Ligand | 76.3 | 40.4 | |
| R.m.s. deviations | | | |
| Bond lengths (Å) | 0.003 | 0.004 | |
| Bond angles (°) | 0.922 | 0.981 | |
| Validation | | | |
| MolProbity score | 1.10 | 1.69 | |
| Clashscore | 0.63 | 1.37 | |
| Poor rotamers (%) | 1.37 | 4.41 | |
| Ramachandran plot | | | |
| Favored (%) | 95.78 | 95.22 | |
| Allowed (%) | 4.22 | 4.78 | |
| Disallowed (%) | 0.00 | 0.00 | |

**Supplementary Table 2:** System setup for the MD simulations.

| | propionate-bound FFA2 | GLPG0974-bound FFA2 | | | |
|---|---|---|---|---|---|
| | | K65 deprotonated | | K65 protonated | |
| | | pose 1 | pose 2 | pose 1 | pose 2 |
| **initial box dimensions (Å)** | 80 × 77 × 84 | 112 × 111 × 104 | | | |
| **number of atoms** | 49,264 | 111,959 | 111,962 | 111,958 | 111,961 |
| **number of water molecules** | 9,319 | 23,661 | 23,662 | 23,660 | 23,661 |
| **number of POPC molecules** | 125 | 269 | | | |
| **neutralizing NaCl concentration (mM)** | 150 | 100 | | | |

**Source Data: Uncropped gels for SDS-PAGE analysis.**

Uncropped gel for Supplementary Fig. 1c

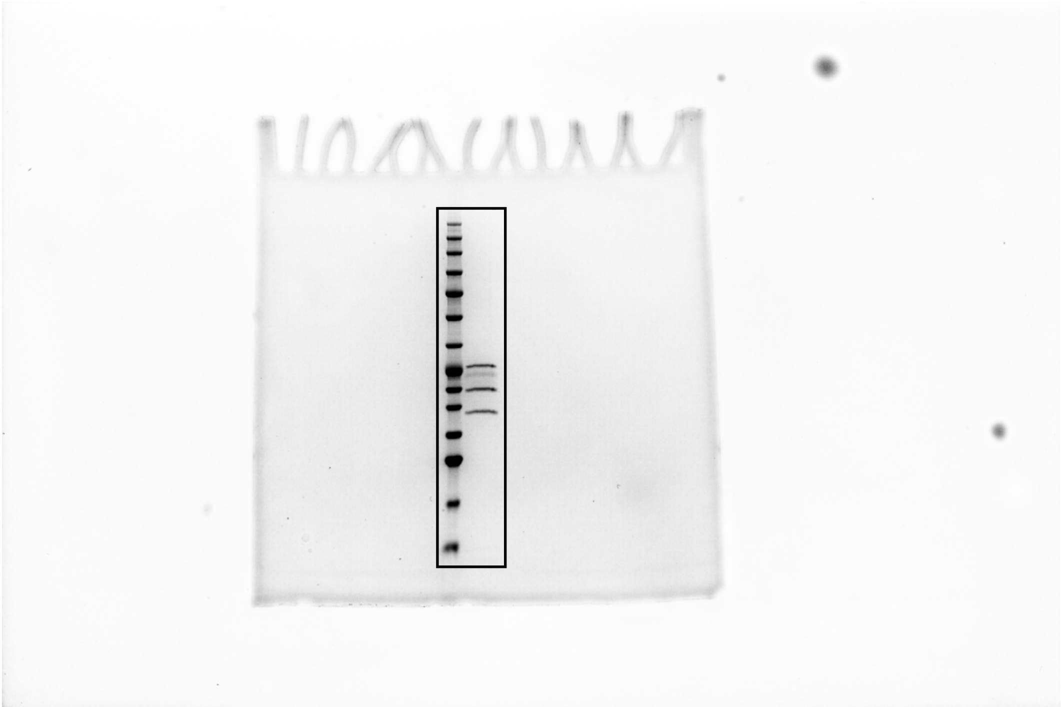

Uncropped gel for Supplementary Fig. 2b

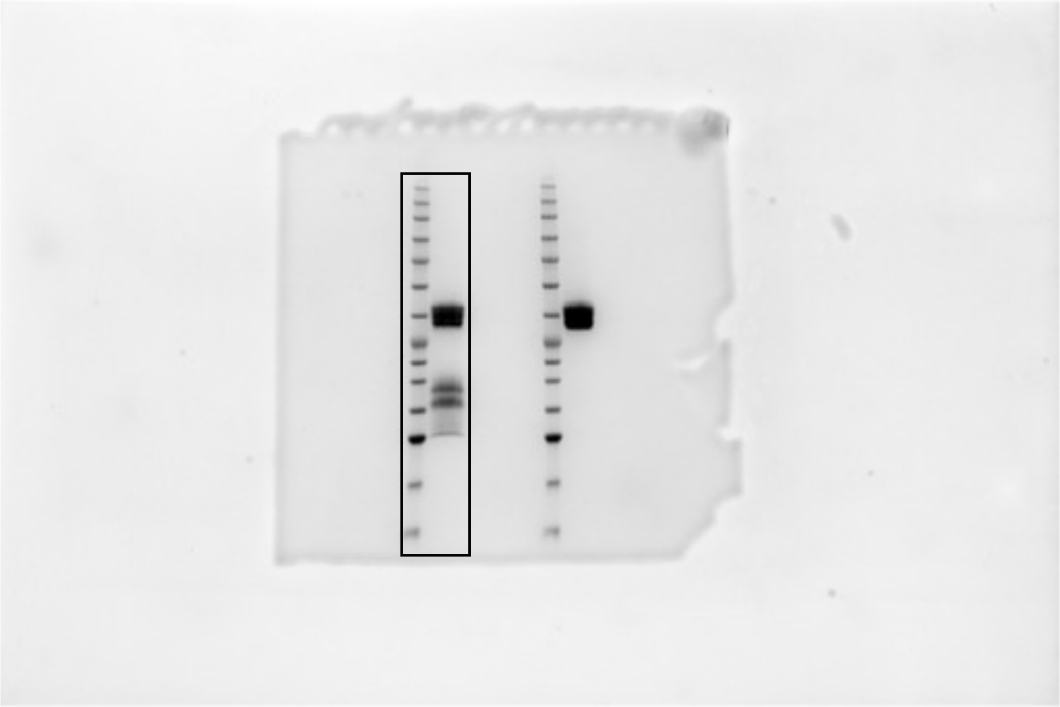

## References


91 Luginina, A. *et al.* Structure-based mechanism of cysteinyl leukotriene receptor inhibition by antiasthmatic drugs. *Sci Adv* **5**, eaax2518, doi:10.1126/sciadv.aax2518 (2019).

92 Xia, A. *et al.* Cryo-EM structures of human GPR34 enable the identification of selective antagonists. *Proc Natl Acad Sci U S A* **120**, e2308435120, doi:10.1073/pnas.2308435120 (2023).

93 Yin, H. *et al.* Insights into lysophosphatidylserine recognition and Galpha(12/13)-coupling specificity of P2Y10. *Cell Chem Biol* **31**, 1899-1908 e1895, doi:10.1016/j.chembiol.2024.08.005 (2024).

94 Liang, J. *et al.* Structural basis of lysophosphatidylserine receptor GPR174 ligand recognition and activation. *Nat Commun* **14**, 1012, doi:10.1038/s41467-023-36575-0 (2023).

95 Fan, W. *et al.* Molecular basis for the activation of PAF receptor by PAF. *Cell Rep* **43**, 114422, doi:10.1016/j.celrep.2024.114422 (2024).

96 Chen, H., Huang, W. & Li, X. Structures of oxysterol sensor EBI2/GPR183, a key regulator of the immune response. *Structure* **30**, 1016-1024 e1015, doi:10.1016/j.str.2022.04.006 (2022).

97 Xia, R. *et al.* Structural insight into GPR55 ligand recognition and G-protein coupling. *Cell Res*, doi:10.1038/s41422-024-01044-w (2024).

98 Liu, H. *et al.* Molecular basis for lipid recognition by the prostaglandin D(2) receptor CRTH2. *Proc Natl Acad Sci U S A* **118**, doi:10.1073/pnas.2102813118 (2021).

99 Wang, J. L. *et al.* Functional screening and rational design of compounds targeting GPR132 to treat diabetes. *Nat Metab* **5**, 1726-1746, doi:10.1038/s42255-023-00899-4 (2023).

100 Yang, J. *et al.* Structure-guided discovery of bile acid derivatives for treating liver diseases without causing itch. *Cell* **187**, 7164-7182 e7118, doi:10.1016/j.cell.2024.10.001 (2024).

101 Yu, L. *et al.* Structural insights into sphingosine-1-phosphate receptor activation. *Proc Natl Acad Sci U S A* **119**, e2117716119, doi:10.1073/pnas.2117716119 (2022).

102 Chen, H. *et al.* Structure of S1PR2-heterotrimeric G(13) signaling complex. *Sci Adv* **8**, eabn0067, doi:10.1126/sciadv.abn0067 (2022).

103 Zhao, C. *et al.* Structural insights into sphingosine-1-phosphate recognition and ligand selectivity of S1PR3-Gi signaling complexes. *Cell Res* **32**, 218-221, doi:10.1038/s41422-021-00567-w (2022).

104 Yuan, Y. *et al.* Structures of signaling complexes of lipid receptors S1PR1 and S1PR5 reveal mechanisms of activation and drug recognition. *Cell Res* **31**, 1263-1274, doi:10.1038/s41422-021-00566-x (2021).

105 Hua, T. *et al.* Activation and Signaling Mechanism Revealed by Cannabinoid Receptor-G(i) Complex Structures. *Cell* **180**, 655-665 e618, doi:10.1016/j.cell.2020.01.008 (2020).

106 Xu, P. *et al.* Structural identification of lysophosphatidylcholines as activating ligands for orphan receptor GPR119. *Nat Struct Mol Biol* **29**, 863-870, doi:10.1038/s41594-022-00816-5 (2022).

107 Liu, H. *et al.* Structural insights into ligand recognition and activation of the medium-chain fatty acid-sensing receptor GPR84. *Nat Commun* **14**, 3271, doi:10.1038/s41467-023-38985-6 (2023).

108 Wang, N. *et al.* Structural basis of leukotriene B4 receptor 1 activation. *Nat Commun* **13**, 1156, doi:10.1038/s41467-022-28820-9 (2022).

109 Qu, C. *et al.* Ligand recognition, unconventional activation, and G protein coupling of the prostaglandin E(2) receptor EP2 subtype. *Sci Adv* **7**, doi:10.1126/sciadv.abf1268 (2021).

110 Huang, S. M. *et al.* Single hormone or synthetic agonist induces G(s)/G(i) coupling selectivity of EP receptors via distinct binding modes and propagating paths. *Proc Natl Acad Sci U S A* **120**, e2216329120, doi:10.1073/pnas.2216329120 (2023).

111 Nojima, S. *et al.* Cryo-EM Structure of the Prostaglandin E Receptor EP4 Coupled to G Protein. *Structure* **29**, 252-260 e256, doi:10.1016/j.str.2020.11.007 (2021).

112 Wu, C. *et al.* Ligand-induced activation and G protein coupling of prostaglandin F(2alpha) receptor. *Nat Commun* **14**, 2668, doi:10.1038/s41467-023-38411-x (2023).

113 Wang, J. J. *et al.* Molecular recognition and activation of the prostacyclin receptor by anti-pulmonary arterial hypertension drugs. *Sci Adv* **10**, eadk5184, doi:10.1126/sciadv.adk5184 (2024).

114 Li, X. *et al.* Structural basis for ligand recognition and activation of the prostanoid receptors. *Cell Rep* **43**, 113893, doi:10.1016/j.celrep.2024.113893 (2024).

115 Xiong, Y. *et al.* Identification of oleic acid as an endogenous ligand of GPR3. *Cell Res*, doi:10.1038/s41422-024-00932-5 (2024).
