# Supplementary material for: Structural insights into lipid chain-length selectivity and allosteric regulation of FFA2
Source: Nat Commun. 2025 Mar 26;16:2809. doi: 10.1038/s41467-025-57983-4 (PMC11947310; doi:10.1038/s41467-025-57983-4)
Supplement: Supplementary file 2 — Reporting Summary [file 41467_2025_57983_MOESM2_ESM.pdf]

nature portfolio

Corresponding author(s): Hideaki E. Kato

Last updated by author(s): Mar 18, 2024

Reporting Summary

Nature Portfolio wishes to improve the reproducibility of the work that we publish. This form provides structure for consistency and transparency in reporting. For further information on Nature Portfolio policies, see our Editorial Policies and the Editorial Policy Checklist.

Statistics

For all statistical analyses, confirm that the following items are present in the figure legend, table legend, main text, or Methods section.

n/a	Confirmed	
☐	☒	The exact sample size (n) for each experimental group/condition, given as a discrete number and unit of measurement
☐	☒	A statement on whether measurements were taken from distinct samples or whether the same sample was measured repeatedly
☐	☒	The statistical test(s) used AND whether they are one- or two-sided<br>*Only common tests should be described solely by name; describe more complex techniques in the Methods section.*
☒	☐	A description of all covariates tested
☐	☒	A description of any assumptions or corrections, such as tests of normality and adjustment for multiple comparisons
☐	☒	A full description of the statistical parameters including central tendency (e.g. means) or other basic estimates (e.g. regression coefficient) AND variation (e.g. standard deviation) or associated estimates of uncertainty (e.g. confidence intervals)
☐	☒	For null hypothesis testing, the test statistic (e.g. F, t, r) with confidence intervals, effect sizes, degrees of freedom and P value noted<br>*Give P values as exact values whenever suitable.*
☒	☐	For Bayesian analysis, information on the choice of priors and Markov chain Monte Carlo settings
☒	☐	For hierarchical and complex designs, identification of the appropriate level for tests and full reporting of outcomes
☒	☐	Estimates of effect sizes (e.g. Cohen's d, Pearson's r), indicating how they were calculated

Our web collection on statistics for biologists contains articles on many of the points above.

Software and code

Policy information about availability of computer code

Data collection	EPU, SoftMax Pro
Data analysis	RELION 4.0, CryoSPARC v4.3.0 & v4.4.1, COOT 0.9.8.92, Servalcat 0.4.39, UCSF Chimera X 1.6.1, GraphPad Prism 10, Flow Jo, CHARMM ver 35b2, JAGUAR ver 7.9, MEAD ver 2.2.9, Karlsberg ver 1.0.2, VMD ver 1.9.2, NAMD ver 2.13, Maestro ver 2022-3, PPM 3.0, Dabble ver 2.7.9, VMD ver 1.9.4a57, vmd-python ver 3.0.6

For manuscripts utilizing custom algorithms or software that are central to the research but not yet described in published literature, software must be made available to editors and reviewers. We strongly encourage code deposition in a community repository (e.g. GitHub). See the Nature Portfolio guidelines for submitting code & software for further information.

Data

Policy information about availability of data

All manuscripts must include a data availability statement. This statement should provide the following information, where applicable:

- Accession codes, unique identifiers, or web links for publicly available datasets
- A description of any restrictions on data availability
- For clinical datasets or third party data, please ensure that the statement adheres to our policy

The raw images of TUG-1375/4-CMTB-bound FFA2–Gi complex and GLPG0974-bound FFA2-BRIL before motion correction have been deposited in the Electron

Microscopy Public Image Archive under accession EMPIAR-12493. Atomic coordinates for the TUG-1375/4-CMTB-bound FFA2-Gi complex and GLPG0974-bound FFA2-BRIL have been deposited in the Protein Data Bank under accession code 8Y6W and 8Y6Y. The associated electron microscopy data have been deposited in the Electron Microscopy Data Bank under accession code EMD-39003 and EMD-39004. The simulation data for propionate-bound FFA2 and GLPG0974-bound FFA2-BRIL have been deposited on Zenodo: entry 14853893 and 14885834. All other data provided with this paper.

Research involving human participants, their data, or biological material

Policy information about studies with human participants or human data. See also policy information about sex, gender (identity/presentation), and sexual orientation and race, ethnicity and racism.

Reporting on sex and gender	No concern about this section in this study.
Reporting on race, ethnicity, or other socially relevant groupings	No concern about this section in this study.
Population characteristics	No concern about this section in this study.
Recruitment	No concern about this section in this study.
Ethics oversight	No concern about this section in this study.

Note that full information on the approval of the study protocol must also be provided in the manuscript.

Field-specific reporting

Please select the one below that is the best fit for your research. If you are not sure, read the appropriate sections before making your selection.

☒ Life sciences ☐ Behavioural & social sciences ☐ Ecological, evolutionary & environmental sciences

For a reference copy of the document with all sections, see nature.com/documents/nr-reporting-summary-flat.pdf

Life sciences study design

All studies must disclose on these points even when the disclosure is negative.

Sample size	For signaling studies, we used a sample size of 3-5 to enable repeatability and to control for biological variance typical in biochemical assays, with a minimum of two measurements per tested concentration. For molecular dynamics simulations of propionate and GLPG0974, 12 and 3 independent simulations were performed, respectively, with initial atom velocities chosen randomly and independently.
Data exclusions	No data was excluded from the analysis.
Replication	Cell-based experiments were independently performed at least three times. We verify that all experiments were successfully performed in duplicate or triplicate.
Randomization	For cryo-EM studies, particles were randomly assigned to half-maps for resolution determination. Randomization was not relevant to the other experiments in our study as these assays don't have unknown covariates.
Blinding	Blinding was not relevant to the experiments in our study since no subjective allocation was involved in our study.

Reporting for specific materials, systems and methods

We require information from authors about some types of materials, experimental systems and methods used in many studies. Here, indicate whether each material, system or method listed is relevant to your study. If you are not sure if a list item applies to your research, read the appropriate section before selecting a response.

Materials & experimental systems

n/a	Involved in the study	
☐	☒	Antibodies
☐	☒	Eukaryotic cell lines
☒	☐	Palaeontology and archaeology
☒	☐	Animals and other organisms
☒	☐	Clinical data
☒	☐	Dual use research of concern
☒	☐	Plants

Methods

n/a	Involved in the study	
☒	☐	ChIP-seq
☐	☒	Flow cytometry
☒	☐	MRI-based neuroimaging

Antibodies

Antibodies used	anti-Flag epitope (DYKDDDDK) tag mouse monoclonal antibody (Clone 1E6, FujiFilm Wako Pure Chemicals, cat no. 012-22384); goat anti-mouse IgG secondary antibody conjugated with Alexa Fluor 488 (Thermo Fisher Scientific, cat no. A11001); Anti-BRIL Fab BAG2, an affinity-matured synthetic antibody that binds to the BRIL sequence. Its sequence was acquired from the published paper in which it was reported (https://www.nature.com/articles/s41467-020-15363-0).
Validation	All the commercial antibodies were verified by the manufactures according to immunoblots and images on their websites. These were validated by their respective manufacturers as indicated at these links: https://labchem-wako.fujifilm.com/jp/product/detail/W01W0101-2238.html https://www.thermofisher.com/antibody/product/Goat-anti-Mouse-IgG-H-L-Cross-Adsorbed-Secondary-Antibody-Polyclonal/A-11001 For BAG2, validation of antigen binding was described in the published papers refereneced above.

Eukaryotic cell lines

Policy information about cell lines and Sex and Gender in Research

Cell line source(s)	HEK293A purchased from Thermo Fisher Scientific (cat. no. R70507); HEK293S GnTI- purchased from Thermo Fisher Scientific (cat. no. 50-238-3284); Hi5 purchased from Expression Systems (cat. no. 94-002F)
Authentication	None were authenticated.
Mycoplasma contamination	Hi5 and HEK293S cells were not tested for mycoplasma contamination. HEK293A cells were regularly screened to ensure the absence of mycoplasma contamination using MycoAlert Mycoplasma detection kit (Lonza).
Commonly misidentified lines (See ICLAC register)	None of commonly misidentified lines were used in this study.

Plants

Seed stocks	No concern about this section in this study.
Novel plant genotypes	No concern about this section in this study.
Authentication	No concern about this section in this study.

Flow Cytometry

Plots

Confirm that:

- [ ] The axis labels state the marker and fluorochrome used (e.g. CD4-FITC).
- [ ] The axis scales are clearly visible. Include numbers along axes only for bottom left plot of group (a 'group' is an analysis of identical markers).
- [ ] All plots are contour plots with outliers or pseudocolor plots.
- [ ] A numerical value for number of cells or percentage (with statistics) is provided.

Methodology

Sample preparation	HEK293A cells were transfected by combining 3 µL of polyethylenimine Max solution (1mg mL-1) and 200 ng of a plasmid encoding FLAG epitope-tagged GPCR.
Instrument	EC800 flow cytometer (sony)
Software	FlowJo 10 (FlowJo), Prism 10 (GraphPad)
Cell population abundance	N/A

Gating strategy

Live cells were gated with a forward scatter (FS-Peak-Lin) cutoff of 390 setting a gain value of 1.7.

☐ Tick this box to confirm that a figure exemplifying the gating strategy is provided in the Supplementary Information.
